# OP45 Incorporation Of The Tetravalent Dengue Vaccine In The Brazilian Public Health System: A Cost–Benefit Analysis

**DOI:** 10.1017/S0266462324001077

**Published:** 2025-01-07

**Authors:** Ana Zulmira Gomes dos Santos, Gabriella Santos Barros, Gabriela Bittencourt Gonzalez Mosegui, Gabriel Rodrigues Martins de Freitas

## Abstract

**Introduction:**

Dengue, a mosquito-borne disease, is prevalent in Brazil, ranking third in the Americas. The country has confirmed all four serotypes (DENV-1, DENV-2, DENV-3, and DENV-4) of the virus. In 2023, a tetravalent vaccine, approved for individuals aged four to 60 years, opens the possibility of integration into the public health system.

**Methods:**

The invested amount for purchasing two doses of the vaccine to immunize the entire eligible population in the year 2023 was calculated. A comprehensive analysis of direct and indirect costs was conducted regarding the integration of the tetravalent dengue virus vaccine into Brazil’s public healthcare system. Direct costs encompassed treatment expenses for mild, moderate, and severe cases, while indirect costs involved workdays lost and mortality-related expenses. Direct and indirect costs were compared in two scenarios (with and without vaccination), followed by a cost–benefit ratio calculation.

**Results:**

The investment for procuring vaccines for over 168 million Brazilians amounted to approximately EUR17.9 billion, resulting in savings of about EUR193.5 million. Indirect costs were particularly significant when compared to the non-vaccinated population. Considering herd immunity and reducing the vaccinated population to 70 percent of the eligible populace, the invested amount was approximately EUR12.5 billion, while savings reached EUR214.2 million. A cost-benefit ratio calculation revealed a return of one centavo (EUR0.0019) for every BRL1.00 invested (EUR0.0019), and considering herd immunity, the cost–benefit ratio was approximately 0.02.

**Conclusions:**

Despite Brazil being one of the countries with the highest prevalence of dengue in the Americas, the availability of the tetravalent dengue virus vaccine in the country’s public system does not seem to be a sustainable option, given the unfavorable cost–benefit analysis for such implementation. Nevertheless, it is imperative to conduct this analysis with due consideration for alternative scenarios.

